# Machine Learning techniques in breast cancer prognosis prediction: A primary evaluation

**DOI:** 10.1002/cam4.2811

**Published:** 2020-03-10

**Authors:** Carlo Boeri, Corrado Chiappa, Federica Galli, Valentina De Berardinis, Laura Bardelli, Giulio Carcano, Francesca Rovera

**Affiliations:** ^1^ SSD Breast Unit – ASST‐Settelaghi Varese Senology Research Center Department of Medicine University of Insubria Varese Italy

**Keywords:** algorithm, Artificial Neural Network (ANN), breast cancer, predictive models, Support Vector Machine (SVM)

## Abstract

More than 750 000 women in Italy are surviving a diagnosis of breast cancer. A large body of literature tells us which characteristics impact the most on their prognosis. However, the prediction of each disease course and then the establishment of a therapeutic plan and follow‐up tailored to the patient is still very complicated. In order to address this issue, a multidisciplinary approach has become widely accepted, while the Multigene Signature Panels and the Nottingham Prognostic Index are still discussed options. The current technological resources permit to gather many data for each patient. Machine Learning (ML) allows us to draw on these data, to discover their mutual relations and to esteem the prognosis for the new instances. This study provides a primary evaluation of the application of ML to predict breast cancer prognosis. We analyzed 1021 patients who underwent surgery for breast cancer in our Institute and we included 610 of them. Three outcomes were chosen: cancer recurrence (both loco‐regional and systemic) and death from the disease within 32 months. We developed two types of ML models for every outcome (Artificial Neural Network and Support Vector Machine). Each ML algorithm was tested in accuracy (=95.29%‐96.86%), sensitivity (=0.35‐0.64), specificity (=0.97‐0.99), and AUC (=0.804‐0.916). These models might become an additional resource to evaluate the prognosis of breast cancer patients in our daily clinical practice. Before that, we should increase their sensitivity, according to literature, by considering a wider population sample with a longer period of follow‐up. However, specificity, accuracy, minimal additional costs, and reproducibility are already encouraging.

## Graphical abstract

Machine Learning (ML) allows us to discover relations between prognostic factors and to predict breast cancer prognosis. These models might become an additional resource in our daily clinical practice.

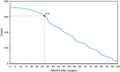

## 1 INTRODUCTION

Breast cancer is the most frequent female malignant neoplasia.^1^ According to the 2017 epidemiological data, more than 50 000 women in 1 year received a diagnosis of breast cancer in Italy and, overall, more than 750 000 women survive a previous diagnosis.^2^ Many scientific papers evaluate the impact of different variables on the prognosis of such a large section of population. Disease‐free and overall survival depend mainly on the molecular biology and on the stage of the disease.^3^ In particular, inflammatory breast cancer and metastatic disease have a deep influence on them.^4^, ^5^ Age, infiltrating cancer, axillary lymph node involvement, tumor size, histological subtype, HER2, Ki67, estrogen and progesterone receptor expression, grading, lymphovascular invasion, multifocality or multicentricity, resection margins, tumor infiltrating lymphocytes are the other main factors described in the literature.^6^, ^7^, ^8^, ^9^, ^10^, ^11^, ^12^, ^13^, ^14^, ^15^, ^16^, ^17^, ^18^, ^19^, ^20^, ^21^ Some statistical methods, such as multivariate regression, allow us to know not only the importance of each variable, but also how these relate to each other defining the disease evolution.

However, even though this information is helpful for us to know the behavior of breast cancer, the prediction of each patient's prognosis and then the establishment of a specific therapeutic plan and follow‐up is still very difficult.^22^ Thereby, this purpose belongs to Breast Units since their foundation. Multidisciplinary approach, Multigene Signature Panels (MSPs), Nottingham Prognostic Index (NPI) are three relevant examples. The multidisciplinary approach implies that the therapeutic plan suggested to each patient is determined combining the evaluation of different specialists, such as breast surgeons, oncologists, radiation therapists, nuclear medicine physicians, and pathologists. MSPs, such as MammaPrint or Oncotype DX, conduce a risk classification for cancer recurrence in order to identify the cases that could really benefit from chemotherapy, especially in case of nonmetastatic and luminal cancers.^23^, ^24^, ^25^ The MSPs’ purpose is to reduce unnecessary treatments, to avoid toxicity and to minimize costs. Nevertheless, the execution of these tests leads to remarkable costs and they are applicable only in extremely selected cases.^26^, ^27^ Last but not least, NPI is a prognostic score which is based on tumor size, number of metastatic lymph nodes and cancer grade, and asses the survival rate among four different possibilities.^28^

The current technological resources allow us to collect many data for each patient, either clinical, pathological or regarding the follow‐up.^29^ Machine Learning (ML) enables to draw on these data, to discover their mutual relations and to esteem the prognosis for new instances.^30^, ^31^ Therefore, they can learn from previous patients and apply this knowledge to predict autonomously the course of the disease of the present ones.^32^ The Artificial Neural Network (ANN) and the Support Vector Machine (SVM) are two major ML methods, able to categorize subjects into different classes according to the risk of recurrence or death.^33^ ANN’s structure looks like a biological neural network, in which there are neurons and connections between them. In the ANN neurons are placed in different layers: an input level, an output level, and one or more intermediate levels.^32^ Every unit receives simultaneously inputs by different neurons of the previous levels. According to the intensity of the stimulus received, each neuron produces itself a signal toward the neurons of the following layer, up to the output layer, which is the last one.^34^, ^35^ SVM builds a hyperplane that separates data mapped according to their characteristics in a high‐ or infinite‐dimensional space.^36^

The scientific development has spread the use of ML in many different fields of science, industry, and finance.^37^ They have been used for web search engines, traffic forecasts, mail filters, and self‐driving vehicles. According to a review conducted by Cruz and Wishart, that analyzed 79 studies, ML may improve by 15%‐25% the accuracy in predicting cancer onset, its recurrence, and its mortality.^38^ An accurate and individualized projection may guide treatment and follow‐up as well as relieve the uncertainties about the future that inevitably belong to the oncologic patients.^22^, ^39^ Compared to MSPs, these techniques cost less and are based on data that are already available. Moreover, they allow to integrate clinical and pathological information.

That being said, this study aims at providing a primary evaluation of the use of ML in our Centre to predict the prognosis in breast cancer.

## 2 METHODS

### 2.1 Subjects and study design

We have analyzed retrospectively 1021 consecutive patients of both genders who underwent surgery for breast cancer in our Institute from April 2008 to December 2016.

We have collected the following variables for each patient: gender; age; menopause; BMI; familiarity; BRCA‐1 or BRCA‐2 gene mutation; comorbidities; previous breast cancer and its characteristics; cTNM; inflammatory breast cancer; neoadjuvant chemotherapy; pathologic response; type of surgery; Radioguided Occult Lesion Localization (ROLL); breast reconstruction; simultaneous surgeries; histological type; in situ component; unifocality, multifocality, or multicentricity; tumor size; extension to the chest wall or the skin; Paget's disease of the nipple; pTNM; staging; grading; lymphovascular invasion; perineural invasion; HER2, Ki67, estrogen and progesterone receptor expression; number of removed axillary lymph nodes; number of involved axillary lymph nodes (ITC, micro‐ and macro‐metastasis); extranodal extension; adjuvant therapy (chemotherapy, hormonal therapy, radiotherapy); follow‐up information (last examination, length of FU, disorders); recurrence or new breast cancer onset (time, site, and treatment); death (time and cause) (Table 1).

**Table 1:** Study population

	Mean ± SD/% (No.)		Mean ± SD/% (No.)
Gender	F = 100% (610)	Age (y)	59.711 ± 12.886
Menopause	70.82% (432)	Menopause age (y)	49.611 ± 4.870
Arterial hypertension	35.08% (214)	Diabetes mellitus	7.70% (47)
Coronary heart disease	4.26% (26)	Previous ovarian cancer	1,15% (7)
BMI (kg/m^2^)	25.765 ± 6.019	Familiarity	27.87% (170)
BRCA mutation	BRCA1 = 1.64% (10)	Chest wall/skin invasion	1.48% (9)
	BRCA2 = 0.49% (3)		
cT	x = 2.95% (18)	pT	0 = 1.64% (10)
	0 = 0.49% (3)		is = 0.82% (5)
	is = 1.8% (11)		1 = 62.3% (380)
	1 = 59.18% (361)		2 = 31.15% (190)
	2 = 29.67% (181)		3 = 2.62% (16)
	3 = 2.79% (17)		4 = 1.48 (9)
	4 = 3.11% (19)		
cN	x = 0.49% (3)	pN	x = 1.64% (10)
	0 = 82.46% (503)		0 = 63.11% (385)
	1 = 14.75% (90)		0(i+)= 1.64% (10)
	2 = 0.82% (5)		1mi = 6.07% (37)
	3 = 1.48% (9)		1 = 17.05% (104)
			2 = 6.89% (42)
			3 = 5.08% (31)
M	0 = 98.03% (598)	Inflammatory breast cancer	0.98% (6)
	1 = 1.97% (12)		
Clinical stage	0 = 1.97% (12)	Pathologic stage	0 = 1.31% (8)
	IA = 54.1% (330)		IA = 47.54% (290)
	IIA = 24.92% (152)		IB = 4.26% (26)
	IIB = 7.54% (46)		IIA = 26.39% (161)
	IIIA = 1.8% (11)		IIB = 7.70% (47)
	IIIB = 2.3% (14)		IIIA = 6.72% (41)
	IIIC = 1.31% (8)		IIIB = 0.98% (6)
	IV = 1.8% (11)		IIIC = 4.59% (28)
			IV = 1.97% (12)
Neoadjuvant chemotherapy	7.7% (47)	Pathologic response after neoadjuvant chemotherapy	None 1.48% (9)
			Partial 4.75% (29)
			Complete 1.31% (8)
Tumor size (pathologic size of the major nodule) (cm)	2.016 ± 1.466	Focality	Unifocal 80.66% (492)
			Multifocal 9.02% (55)
			Multicentric 8.85% (54)
Histological type	DCI NST 77.7% (474)	Type of surgery	Breast‐conserving 61.64% (376)
	DCI special type 7.7% (47)		
			Mastectomy 38.36% (234)
	LCI 10.33% (63)		
	Mixed types 2.46% (15)		
SLNB	79.67% (486)	ALND	35.41% (216)
No. of removed LNs	8.138 ± 8.917	No. of metastatic LNs	1.495 ± 3.814
Lymphovascular invasion	18.36% (112)	Neuroinvasion	4.92% (30)
Extranodal extension	6.72% (41)	Grade	G1 = 4.75% (29)
			G2 = 63.11% (385)
			G3 = 30.82% (188)
ER (%)	85.742 ± 31.865	PgR (%)	57.324 ± 39.878
Ki67 (%)	25.358 ± 17.465	p53 (%)	12.995 ± 26.729
HER2	12.13% (74)	Adjuvant chemotherapy	41.48% (253)
Adjuvant radiotherapy	71.64% (437)	Adjuvant hormonal therapy	83.61% (510)
Follow‐up (mos)	61.302 ± 22.757	New contralateral breast cancer	0.32% (2)
Loco‐regional recurrence within 32 mo	2.95% (18)	Systemic recurrence within 32 mo	4.1% (25)
Death from breast cancer within 32 mo	3.44% (21)		

Ninety‐two (92) patients were excluded from our sample because of incomplete data. We excluded also males (4), those with a previous breast cancer in any side (80), cases of bilateral cancer (70), those affected by cancer in situ (60), and those who underwent surgery within the last 32 months (105). Overall, 610 female patients were considered. The 12 patients diagnosed since the beginning with a Stage IV disease were not considered to predict recurrence, because they were not disease‐free after the treatment. However, these patients were included in the sample used to predict death from breast cancer.

All the subjects included in the study previously authorized the collection and the processing of their personal data through an informed consent. This was a pretrial and retrospective study that did not affect in any way the treatment of each patient. Therefore, the authors did not undergo yet an ethics committee consultation that would be highly recommended before using ML algorithms in the clinical practice.

### 2.2 Outcomes

The outcomes of the predictive models were cancer recurrence (both loco‐regional and systemic) and death from breast cancer. Cancer recurrence was intended as the return of the neoplasia after treatment and after a period in which it could not be detected.^40^ Loco‐regional recurrence refers to a resurgence of the disease in the breast, the chest wall, or the regional lymph nodes defined by the N indicator of the AJCC’s TNM staging system.^41^ The systemic recurrence was the resurgence of the disease in long‐distance lymph nodes or other organs, according to the M indicator.^41^ The two types of recurrence are associated with different risk factors and survival curves and involve different therapeutic approaches.^41^, ^42^

**Figure 1:**
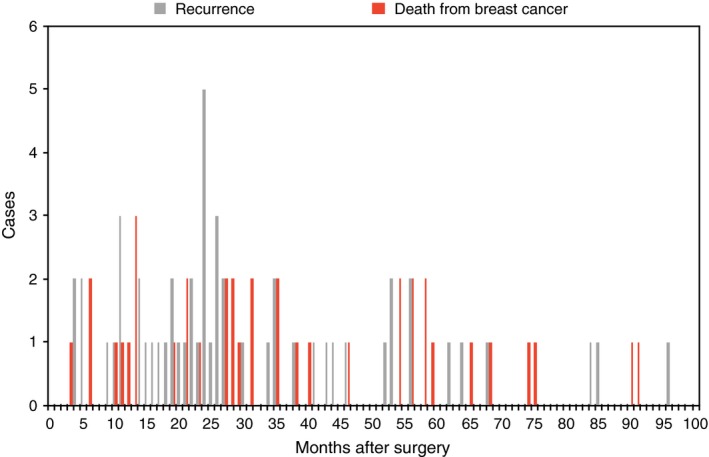
Cases of recurrence and death from breast cancer in 100 mo

**Figure 2:**
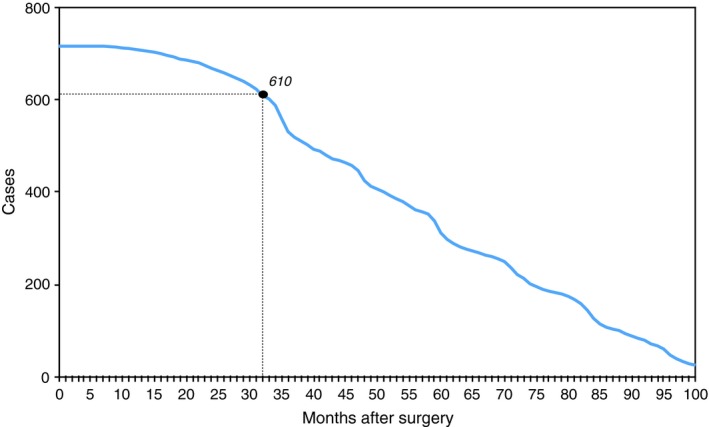
Length of the follow‐up

The follow‐up period was of 32 months. The consideration of a longer period of time would have meant the exclusion from the study of more patients than those 105 excluded. Thirty‐two months was a threshold arbitrarily defined in order to include an adequate number of subjects in the sample, but at the same time, to observe the patients when the risk of recurrence is higher, about 24 months after the cancer was diagnosed^43^, ^44^ (Figures 1 and 2).

### 2.3 ML models’ establishment and statistical analysis

ML models were developed by using the IBM SPSS Modeler 18.1 software. Two types of algorithms (ANN and SVM) were established with the same procedure for each of the three endpoints. The data sample was partitioned each time in three subsamples through randomization. The first subsample was retained while the other two subsamples formed the “training set”. The training set was used to create an SVM and an ANN. The retained subsample (“testing set”) was used at a later stage to test the accuracy, the sensitivity, the specificity and the area under the curve (AUC) of the algorithms. These ML models were then discarded, the second subsample became the testing set and the other two subsamples formed the training set of a new ANN and SVM (*k*‐fold cross validation). After this was repeated once more, all observations were used for both training and validation at the end, once for validation and twice for training the models. Each time, before establishing the models, the minority class of the training set only was oversampled through the Synthetic Minority Over‐sampling Technique (SMOTE) in order to balance the sample. Variables used as inputs were selected by consulting the literature on the most important prognostic factors and confirmed through logistic regression (Table 2).

**Table 2:** Models’ inputs

Loco‐regional recurrence	Systemic recurrence	Death from disease
Tumor size (cm)	Tumor size (cm)	Tumor size (cm)
No. of metastatic axillary lymph nodes (0/1/2/3/…)	No. of metastatic axillary lymph nodes (0/1/2/3/…)	No. of metastatic axillary lymph nodes (0/1/2/3/…)
Estrogen receptor expression (ER%)	Estrogen receptor expression (ER%)	Estrogen receptor expression (ER%)
Grading (G1/G2/G3)	Ki67 expression (%)	Metastatic disease (M = 1/M = 0)
Multicentricity	Multicentricity	Grading (G1/G2/G3)
Skin or chest wall invasion	Pathologic response after neoadjuvant chemotherapy (complete/partial/none)	Pathologic response after neoadjuvant chemotherapy (complete/partial/none)
Adjuvant hormonal therapy	—	—

## 3 RESULTS

### 3.1 Loco‐regional recurrence

**Figure 3:**
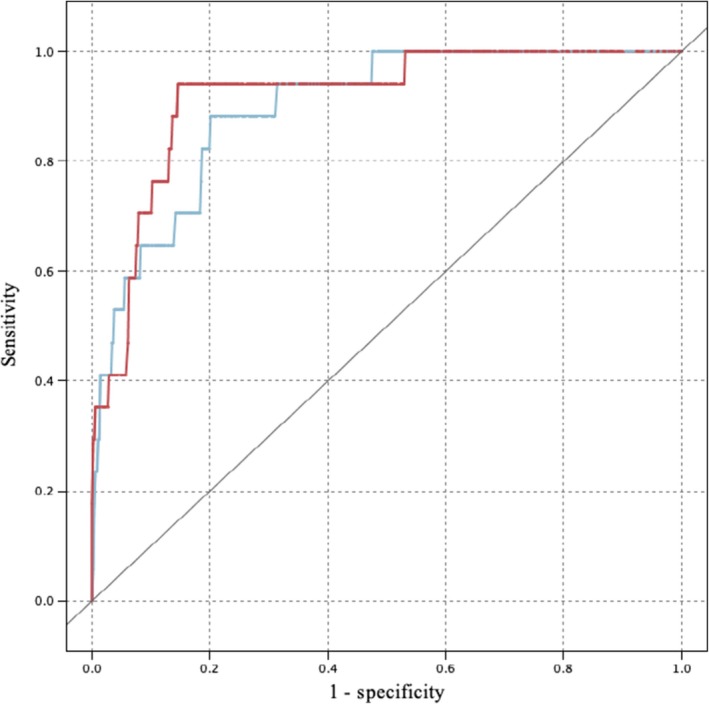
ROC curves for loco‐regional recurrence. Blue line = SVM; Red line = Artificial Neural Network Source: SPSS Modeler.

Twenty‐four cases could not be predicted, because their immunohistochemical profile (ER expression) was not expressed as a percentage. Only one among them had a loco‐regional recurrence in reality. ANN and SVM predicted respectively 6 and 7 cases of loco‐regional recurrence of the 17 reported (ANN sensitivity = 0.35, SVM sensitivity = 0.41). The two models were more specific (ANN specificity = 0.98, SVM specificity = 0.99), since they predicted 546 and 549 cases of no recurrence out of the 557 occurred. Overall, accuracy was respectively 96.17% and 96.86% (Table 3, Figure 3).

**Table 3:** Loco‐regional recurrence

Models	Accuracy	Sensitivity	Specificity	AUC
Artificial Neural Network (ANN)	96.17% (552/574)	0.35 (6/17)	0.98 (546/557)	0.916
Support Vector Machine (SVM)	96.86% (556/574)	0.41 (7/17)	0.99 (549/557)	0.896

### 3.2 Systemic recurrence

**Figure 4:**
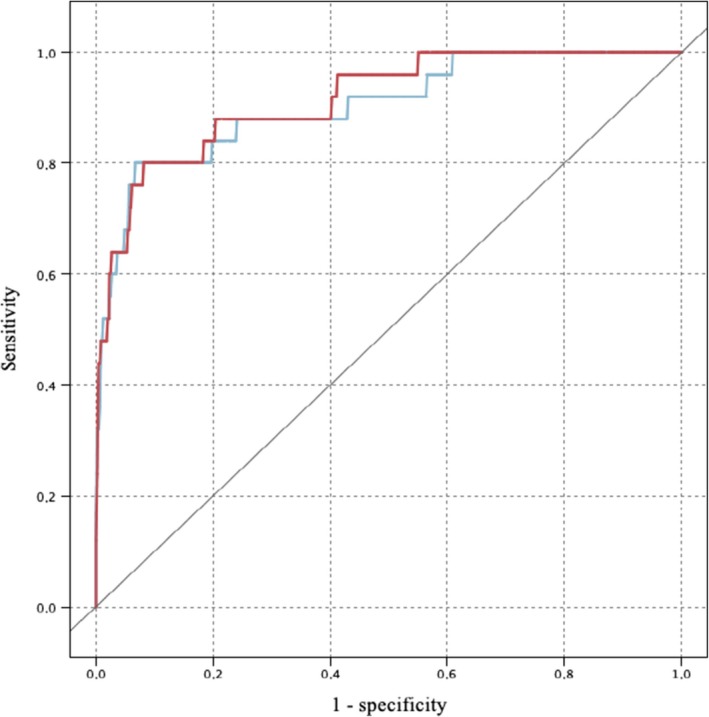
ROC curves for systemic recurrence. Blue line = SVM; red line = Artificial Neural Network Source: SPSS Modeler.

Twenty‐five cases could not be analyzed because they lacked the percentage values of the immunohistochemical profile (ER and Ki67 expression). We did not observe systemic recurrence in any of these 25 patients in reality. ANN and SVM predicted respectively 16 and 14 systemic recurrences out of the 25 really observed (ANN sensitivity = 0.64, SVM sensitivity = 0.56). Among the 548 cases of no recurrence, 530 and 534 were, respectively, esteemed by the two algorithms (ANN and SVM specificity = 0.97). Accuracy was respectively 95.29% and 95.64% (Table 4, Figure 4).

**Table 4:** Systemic recurrence

Models	Accuracy	Sensitivity	Specificity	AUC
Artificial Neural Network (ANN)	95.29% (546/573)	0.64 (16/25)	0.97 (530/548)	0.914
Support Vector Machine (SVM)	95.64% (548/573)	0.56 (14/25)	0.97 (534/548)	0.903

### 3.3 Death from breast cancer

**Figure 5:**
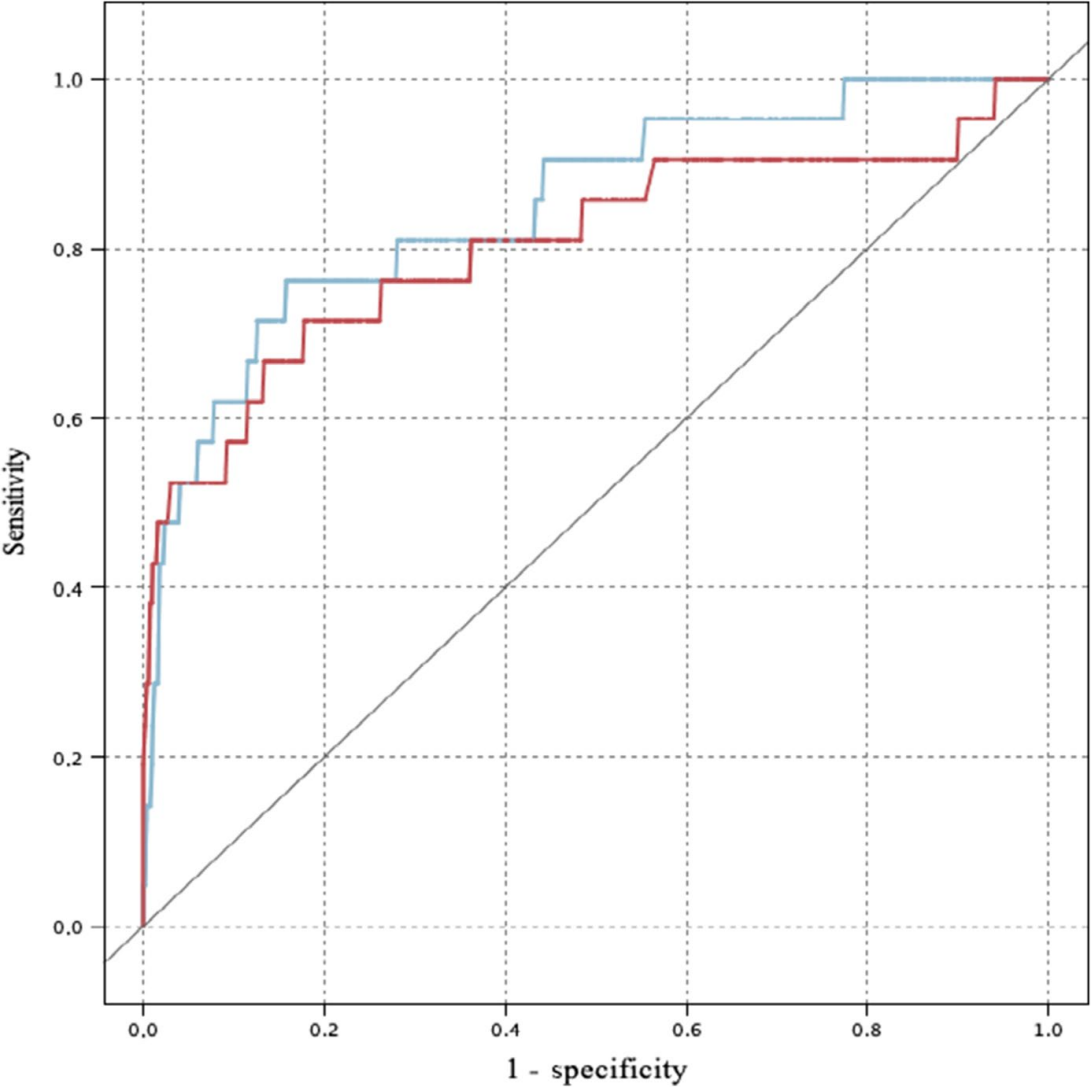
ROC curves for death from breast cancer. Blue line = SVM; red line = Artificial Neural Network Source: SPSS Modeler.

ANN and SVM could not predict 26 cases, because their immunohistochemical profile (ER expression) was not expressed as a percentage. All of them in reality survived more than 32 months from breast cancer. Both ANN and SVM expected 10 cases of death from breast cancer within 32 months of the 21 actually reported (sensitivity = 0.48). In this case as well, ML models were more specific (ANN and SVM specificity = 0.98), since they predicted, respectively, 553 and 549 cases of survival after 32 months among the 563 observed. Accuracy was respectively 96.40% and 95.72% (Table 5, Figure 5).

**Table 5:** Death from breast cancer

Models	Accuracy	Sensitivity	Specificity	AUC
Artificial Neural Network (ANN)	96.40% (563/584)	0.48 (10/21)	0.98 (553/563)	0.804
Support Vector Machine (SVM)	95.72% (559/584)	0.48 (10/21)	0.98 (549/563)	0.849

## 4 DISCUSSION

The ML models developed in this study were able to esteem the prognosis of patients undergoing breast cancer surgery, merging clinical, and pathological information and using only data already available. Additional costs were the software license and the amount of time required to build the models. The purpose of applying ML models in the same institute in which the patients forming the training set were treated was to reduce hidden variables. In doing so, the bias caused by the application of different surgical techniques or dose regimens should have been minimized.

For each of the three output we created an ANN and an SVM. ANN worked properly even with many input variables, due to their layout made of parallel connections. Their major deficiency is their “black box” structure: the intermediate connections cannot be detected nor modified.^32^ SVM is a more recent ML technique, but already used in the oncologic field, including breast cancer.^36^ Its major virtue is its accuracy even when faced with overlapping data, due to the several shapes that the hyperplane can take to split them in different categories.^36^

The six models of ML were able to predict the three outputs with 95.29%‐96.86% accuracy (Tables 3, 4 and 5). Specificity lied between 0.97 and 0.99. However, the sensitivity of these algorithms was low (0.35‐0.64). This might be due to a small population sample observed for a short‐term follow‐up (32 months). If we look at the results of previous studies that applied ML in medicine, we can notice that those models created to predict infrequent events commonly underestimate the minority class in order to be as accurate as possible.^32^, ^38^, ^45^, ^46^, ^47^ This paper confirmed these considerations: very few subjects in the training set got a recurrence or died because of the disease (Table 1). Moreover, the most sensitive ML models of this study were those created to predict systemic recurrence which contained more positive observations in the training set comparing to the other models (Table 4). Therefore, these ML models might improve if trained with a wider population sample and by stretching the length of the follow‐up, since we witness recurrence in breast cancer not only in a short period, but also after a long‐time lag (until 20 years since it was diagnosed).^32^, ^48^, ^49^, ^50^ Thereby, a wider population sample with a longer period of follow‐up would show more cases of recurrence and death both in absolute and relative terms. These adjustments may improve the sensitivity, maybe decreasing specificity, but keeping the same high level of accuracy.^45^, ^46^ Therefore, it is to be considered that this is a preliminary study and that the algorithms are not yet ready to be used in clinical practice.

## 5 CONCLUSIONS

This study explored the use of six ML models to predict the prognosis of breast cancer patients treated in our Institute. Both ANN and SVM were accurate and specific to assess an individualized risk of recurrence or death from the disease (Tables 3, 4, and 5). Nevertheless, ANN and SVM did not prove an adequate level of sensitivity, except when they predicted systemic recurrence. A step further to mitigate this flaw should be to extend the population sample and the length of the follow‐up. The subsequent goal might be the development of ML models that can be used in the daily clinical practice to esteem the prognosis of the patients treated for breast cancer. Reporting the features listed in the Table 2 into a web page, the physician could quantify the risk of loco‐regional and systemic recurrence as well as the risk of death from breast cancer.^36^ Depending on the outcomes, the clinician might be assisted in the choice of the proper adjuvant therapy and follow‐up in terms of frequency and length. The predictive models may assist but shall not replace the physician recommendations, which are based on the association of scientific evidence and personal experience. Therefore, these techniques might merely be an additional and rather inexpensive resource.

## CONFLICT OF INTEREST

The authors have no conflicts of interest to declare.

## Data Availability

Authors can confirm that all relevant data are included in the article and/or its supplementary information files
